# The role of TGF-β signaling and apoptosis in innate and adaptive immunity in zebrafish: a systems biology approach

**DOI:** 10.1186/s12918-014-0116-0

**Published:** 2014-10-24

**Authors:** Che Lin, Chin-Nan Lin, Yu-Chao Wang, Fang-Yu Liu, Yung-Jen Chuang, Chung-Yu Lan, Wen-Ping Hsieh, Bor-Sen Chen

**Affiliations:** Institute of Communication Engineering, National Tsing Hua University, Hsinchu, 30013 Taiwan; Department of Electrical Engineering, National Tsing Hua University, Hsinchu, 30013 Taiwan; Institute of Biomedical Informatics, National Yang-Ming University, Taipei, 11221 Taiwan; Department of Medical Science and Institute of Bioinformatics and Structural Biology, National Tsing Hua University, Hsinchu, 30013 Taiwan; Department of Life Science and Institute of Molecular and Cellular Biology, National Tsing Hua University, Hsinchu, 30013 Taiwan; Institute of Statistics, National Tsing Hua University, Hsinchu, 30013 Taiwan

**Keywords:** *C. albicans*, Zebrafish, Infection, Protein-protein interaction, Dynamic, Modeling, Immune, Adapt immune

## Abstract

**Background:**

The immune system is a key biological system present in vertebrates. Exposure to pathogens elicits various defensive immune mechanisms that protect the host from potential threats and harmful substances derived from pathogens such as parasites, bacteria, and viruses. The complex immune system of humans and many other vertebrates can be divided into two major categories: the innate and the adaptive immune systems. At present, analysis of the complex interactions between the two subsystems that regulate host defense and inflammatory responses remains challenging.

**Results:**

Based on time-course microarray data following primary and secondary infection of zebrafish by *Candida albicans,* we constructed two intracellular protein–protein interaction (PPI) networks for primary and secondary responses of the host. 57 proteins and 341 PPIs were identified for primary infection while 90 proteins and 385 PPIs were identified for secondary infection. There were 20 proteins in common while 37 and 70 proteins specific to primary and secondary infection. By inspecting the hub proteins of each network and comparing significant changes in the number of linkages between the two PPI networks, we identified TGF-β signaling and apoptosis as two of the main functional modules involved in primary and secondary infection.

Smad7, a member of the inhibitor SMADs, was identified to be a key protein in TGF-β signaling involved in secondary infection only. Indeed, the Smad7-dependent feedback system is related to the TGF-β signaling pathway and the immune response, suggesting that Smad7 may be an important regulator of innate and adaptive immune responses in zebrafish. Furthermore, we found that apoptosis was differentially involved in the two infection phases; more specifically, whereas apoptosis was promoted in response to primary infection, it was inhibited during secondary infection.

**Conclusions:**

Our initial *in silico* analyses pave the way for further investigation into the interesting roles played by the TGF-β signaling pathway and apoptosis in innate and adaptive immunity in zebrafish. Such insights could lead to therapeutic advances and improved drug design in the continual battle against infectious diseases.

**Electronic supplementary material:**

The online version of this article (doi:10.1186/s12918-014-0116-0) contains supplementary material, which is available to authorized users.

## Background

Immunity is the natural capability of the body to resist and defend against invasion by pathogenic microbes. In vertebrates, the immune system can generally be divided into two main categories: the innate immune system and the adaptive immune system [1]. The former is responsible for nonspecific immune responses and serves as the front line for rapid defense against foreign invading pathogens [2]. In contrast to the innate immune system, the adaptive immune system is composed of highly specialized systemic cells and defensive processes that are capable of preventing, or at least restricting, specific pathogen invasion. The most significant difference between the innate and adaptive response is that adaptive immunity results in the formation of immunological memory after an initial response to a specific pathogen, leading to an enhanced immune response upon subsequent exposure to the same pathogen.

The zebrafish (*Danio rerio*) has become a powerful model organism for biomedical research in recent years because if its high reproductive rate and low maintenance cost [3]. Indeed, the use of zebrafish to study immunity against infectious disease, including those due to bacterial or viral infections, is rapidly increasing [4,5]. Importantly, zebrafish possesses both innate and adaptive immune systems, making it a particularly suitable model organism for investigating immune mechanisms in vertebrates and mammals [4].

*Candida albicans*, a fungal pathogen that grows as both yeast and filamentous forms, causes opportunistic oral and genital infections in humans [6]. Notably, the ability of *C. albicans* to adapt to diverse environmental changes, including fluctuations in temperature, nutrients, and pH levels, renders it relatively difficult to treat in hosts [7]. Thus, understanding how the zebrafish immune system responds to *C. albicans* infection is crucial to the development of novel therapeutic strategies against infectious diseases in humans.

The transforming growth factor-β (TGF-β) signaling pathway is essential in regulating the immune response to combat infection [8], and defective TGF-β signaling leads to several systemic autoimmune defects. In the canonical pathway, TGF-β signaling mediates its effect through the SMAD pathway [9]. Several studies have revealed that TGF-β signaling suppresses immune responses [8,10,11]. Conversely, however, immune cells can also promote TGF-β signaling [10]. Thus, the molecular mechanisms involved in the TGF-β signaling pathway, which regulates host tolerance as well as innate and adaptive immunity, are important areas for study. Nevertheless, the complex role of TGF-β signaling in maintaining the balance of the immune system remains poorly understood.

In this study, our main objective is to identify the key proteins or functional modules involved in the zebrafish immune response toward primary and secondary infection with *C. albicans*. By constructing two zebrafish intracellular PPI networks for primary and secondary infection, we seek to compare and identify the key proteins, molecular processes, and mechanisms between these two infection phases. In particular, we hope to elucidate the roles of TGF-β signaling in innate and adaptive immune responses.

## Results

### Strategy

**Figure 1:**
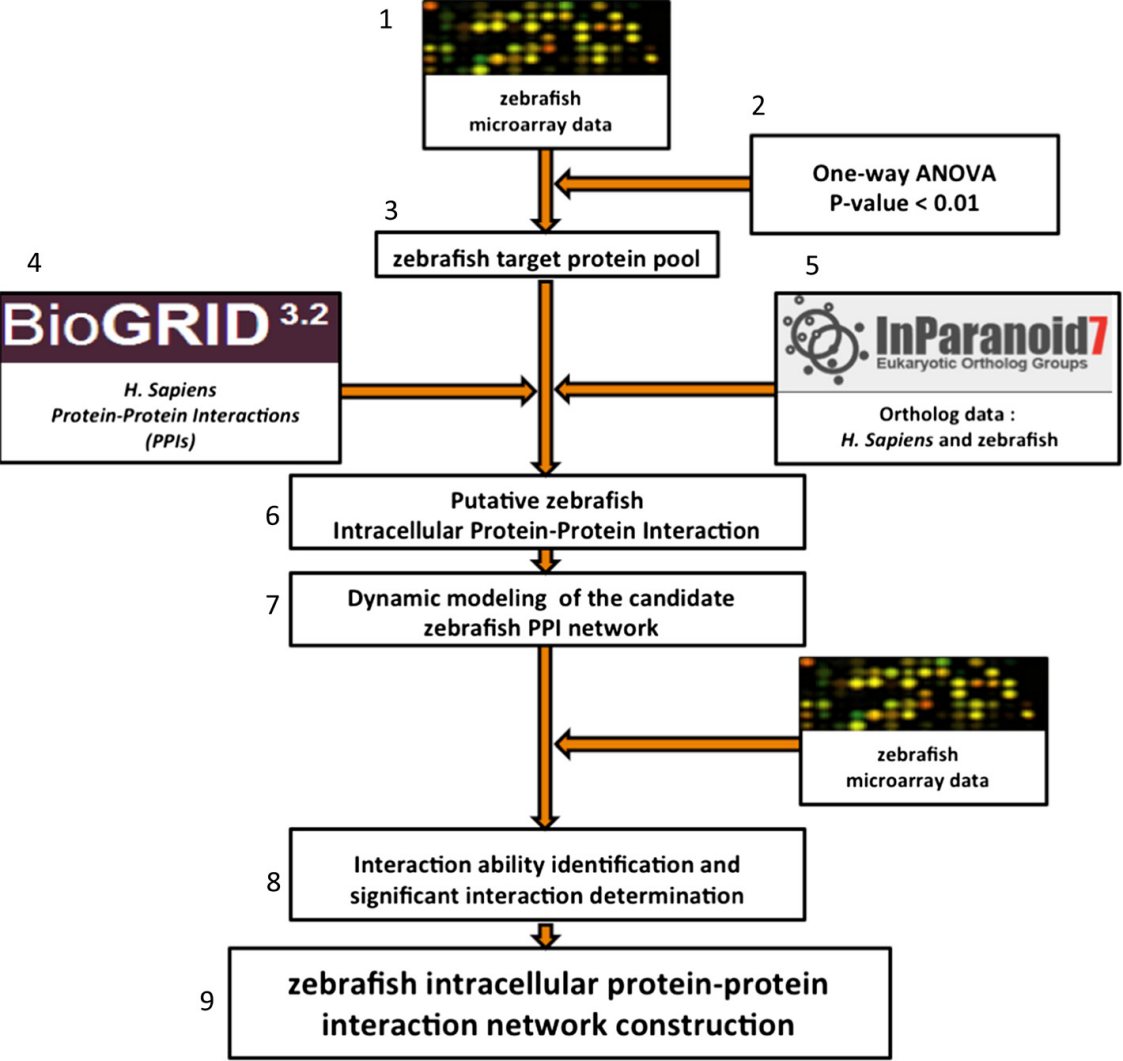
**Flow chart describing the construction of the zebrafish intracellular PPI network.** One-way ANOVA was applied to select the target protein pools. PPIs of *Homo sapiens* data were obtained from the BioGRID database, and orthologous information between zebrafish and *H. sapiens* was extracted from the InParanoid database. By using a dynamic model, we were able to construct the candidate PPI networks for zebrafish. As these candidate PPI networks could not truly represent the intracellular PPIs in zebrafish, we used interaction ability identification to determine the significant interactions. The refined intracellular PPI networks could thus be constructed from these significant interactions.

The method used to construct the dynamic intracellular PPI networks was divided into three key steps: (i) data selection and preprocessing, (ii) selection of the target protein pool, and (iii) construction of the refined PPI networks for zebrafish. The overall framework for the proposed method is depicted in Figure 1.

Our strategy was to collect first all intracellular protein interactions into a candidate intracellular PPI network for zebrafish. The candidate intracellular protein interaction network was further validated and pruned since it could not exactly represent the actual intracellular protein interactions in *C. albicans* infection. A dynamic model was used to describe the candidate intracellular protein interactions. By using microarray data for *C. albicans*-infected zebrafish, the interaction abilities in the dynamic model could thus be determined. Significant PPIs based on these interaction abilities were thus identified to obtain the refined intracellular PPI. The same procedure was applied to construct the zebrafish intracellular PPI network for primary and secondary infection on the basis of time-course microarray data obtained from the corresponding experiments. Details of procedures used to construct the dynamic intracellular PPIs of zebrafish are described in the following sections.

### Dataset selection and target protein pool determination

**Figure 2:**
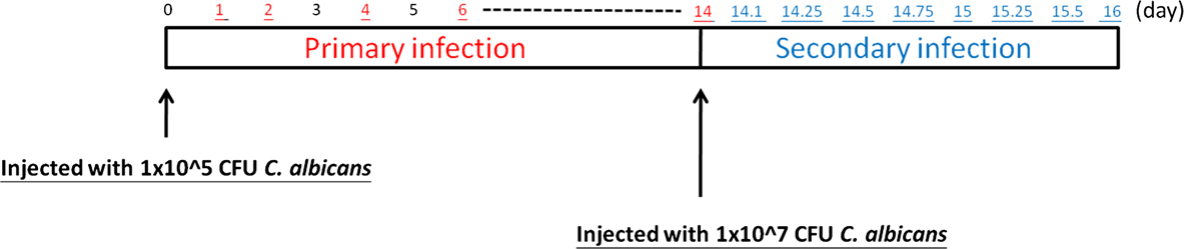
**The bar depicts time points at which data were collected to construct the zebrafish intracellular PPI networks for primary and secondary infection.** For primary infection, zebrafish were injected with 1 × 10^5^ CFU *C. albicans*, and time-course microarray data were collected at 1, 2, 3, 6, and 14 dpi. After 14 days, zebrafish were injected with 1 × 10^7^ CFU *C. albicans*. The time points of the time-course microarray data for secondary infection were 14.1, 14.25, 14.5, 14.75, 15, 15.25, 15.12, and 15.6 dpi.

Three types of data were used in our proposed method: (i) time-course microarray profiles of gene expression of *C. albicans*-infected zebrafish, (ii) PPI data of *H. sapiens*, and (iii) orthologous gene data between zebrafish and *H. sapiens*. There were two sets of time points for primary and secondary *C. albicans* infection for obtaining microarray time-profile data for zebrafish gene expression (primary infection: 1, 2, 3, 6, and 14 dpi; secondary infection: 14.1, 14.25 14.5, 14.75, 15, 15.25, 15.5, and 16 dpi; see Figure 2). Manipulation of the animal model was approved by the Institutional Animal Care and Use Committee of National Tsing Hua University (IRB Approval No. 09808). The PPI data of *H. sapiens* were extracted from the Biological General Repository for Interaction Datasets (BioGRID) database (http://thebiogrid.org/) [12]. The gene ortholog data of zebrafish and humans were obtained from the InParanoid database (http://inparanoid.sbc.su.se/) [13]. For both primary and secondary infection, one-way analysis of variance (ANOVA) was applied to the microarray time-series profile of gene expression to select for differentially expressed proteins. The *p*-value was set at 0.01 with Student t-test for the protein pool selection. Here we treat proteins’ expression as the corresponding genes’ expression and view the gene pool as the protein pool. A total of 422 and 1284 proteins were thus identified as differentially expressed for primary and secondary infection, respectively. After these target protein pools for primary and secondary infection were determined, candidate PPI networks were constructed on basis of the protein pool and PPI information available from data mining. There were a total of 420 and 2312 PPI interactions included in our candidate network by integrating multiple databases (BioGRID and InParanoid7).

### Construction of zebrafish intracellular PPI networks

These candidate PPI networks were obtained by including all available PPI interactions, which cannot truly represent the zebrafish intracellular PPI networks under our experimental settings. Hence, false-positive PPIs were removed on the basis of our experimental data with the help of dynamic modeling of PPI networks.

The dynamic model for the k-th zebrafish target protein in the intracellular PPI network can be represented by a differential equation that follows:

1 $$ {p}_k\left(t+1\right)={p}_k(t)-{K}_k{p}_k(t)+{\displaystyle {\sum}_{m=1}^{M_k}{b}_{km}{p}_k(t){p}_m(t)}+{q}_k+{n}_k(t) $$

where p_k_(t) represents the protein activity level for the k-th zebrafish target protein at time t. We denote M_k_ as the number of PPIs in zebrafish for the k-th target protein; K_k_ denotes the degradation effect for the k-th zebrafish target protein; p_m_[t] denotes the protein activity level for the m-th zebrafish protein that can potentially interact with the k-th target protein, and b_km_ denotes the corresponding interaction ability between the two proteins. The basal level is denoted by q_k_, and the stochastic noise due to model uncertainty and fluctuation of the microarray data is represented by n_k_(t).

The regulatory parameters can be determined with the help of time-course microarray data. To identify the parameters in the model, the gene expression profiles were used to substitute for protein activity levels. Equation (1) can be rewritten in the regression form as follows:

2 $$ {\mathrm{p}}_{\mathrm{k}}\left(\mathrm{t}+1\right)=\left[{\mathrm{p}}_{\mathrm{k}}\left(\mathrm{t}\right){\mathrm{p}}_{\mathrm{k}}\left(\mathrm{t}\right){\mathrm{p}}_1\left(\mathrm{t}\right)\dots {\mathrm{p}}_{\mathrm{k}}\left(\mathrm{t}\right){{\mathrm{p}}_{\mathrm{M}}}_{{}_{\mathrm{k}}}(t)1\right]\left[\begin{array}{l}1-{K}_k\\ {}\kern1em {b}_{\begin{array}{l}k1\\ {}\cdotp \\ {}\cdotp \\ {}\cdotp \end{array}}\\ {}\kern1em {b_k}_{{}_{Mk}}\\ {}\kern1.5em {q}_k\end{array}\right]+{n}_k(t)={\psi}_k(t){\eta}_k+{n}_k(t) $$

Where ψ_k_[t] denotes the regression vector and *η*_k_ denotes the parameter vector for the k-th zebrafish target protein, which is to be estimated. We also used the cubic spline method to interpolate additional time points within the microarray data to avoid over-fitting. Equation (2) for different time points can be rearranged as follows:

3 $$ \left[\begin{array}{l}\mathrm{p}\mathrm{k}\left(\mathrm{t}2\right)\\ {}\mathrm{P}\mathrm{k}\left(\mathrm{t}3\right)\\ {}\vdots \\ {}\mathrm{p}\mathrm{k}\left(\mathrm{t}\mathrm{L}\right)\end{array}\right]=\left[\begin{array}{l}\uppsi \mathrm{k}\left(\mathrm{t}1\right)\\ {}\uppsi \mathrm{k}\left(\mathrm{t}2\right)\\ {}\vdots \\ {}\uppsi \mathrm{k}\left({\mathrm{t}}_{\mathrm{L}\hbox{-} 1}\right)\end{array}\right]\upeta \mathrm{k}+\left[\begin{array}{l}\mathrm{n}\mathrm{k}\left(\mathrm{t}1\right)\\ {}\mathrm{n}\mathrm{k}\left(\mathrm{t}2\right)\\ {}\vdots \\ {}\mathrm{n}\mathrm{k}\left(\mathrm{t}\mathrm{L}-1\right)\end{array}\right] $$

By defining the notations P_k_ = [P_k_(t_2_)] ⋯ p_k_(t_L_)^T^, ψ_k_ = [ψ_k_(t_1_) ⋯ ψ_k_(t_L‐ 1_)]^T^ and Ω_k_ = [n_k_(t_1_) ⋯ n_k_(t_L ‐ 1_)]^T^, equation (3) can then be further rewritten as the linear regression form

4 $$ {P}_k={\psi}_k{\eta}_k+{\varOmega}_k $$

where parameters can be identified by solving a constrained least-squares problem. After the parameters were identified, Akaike’s information criterion (AIC) was used to select significant PPI interactions [14]. The AIC includes both the estimated residual error and model complexity in one statistic. This value increases as the number of parameters increases and decreases as the variance of the residual error decreases. However, the variance of the residual error may decrease with increasing number of parameters. In other words, there exists a tradeoff between estimation accuracy and model complexity. Appropriate model order and significant interactions can be determined by ranking models by increasing AIC. By following these procedures, the intracellular PPI networks for zebrafish were thus constructed.

### Inspection of the constructed zebrafish intracellular PPI networks for primary and secondary infection

We identified 57 proteins and 341 PPIs in the constructed zebrafish intracellular PPI networks during the primary infection, and 90 proteins and 385 PPIs during the secondary infection (see Additional file 1 for the complete networks). A comparison between the two constructed networks indicated that there were 20 proteins that were common to both PPI networks and 37 and 70 proteins that were specific to primary and secondary infection, respectively (see Additional file 1 for complete PPI network lists and figures).

The functions of the 37 proteins that were specific to primary infection were mapped to the following main biological processes: metabolic processes (30.1%), cellular processes (14%), cell communication (10.3%), the cell cycle (8.8%), and the immune response (6.6%). Similarly, the 70 proteins identified to be specific to secondary infection were involved in metabolic processes (26.1%), cellular processes (16.7%), cell communication (13%), developmental processes (9.1%), and transport (5.1%).

### Centrality analysis of the constructed zebrafish intracellular PPI networks for primary and secondary infection

To uncover meaningful implications or insights of the constructed PPI networks, we conducted centrality analysis for both intracellular PPI networks. In particular, we considered three common network centralities, i.e., node degree and betweenness centrality.

For our two constructed zebrafish intracellular PPI networks, 57 and 90 target proteins were identified in primary and secondary *C. albicans* infection, respectively. The top-ranking hub proteins selected by node degree are listed in Tables 1 and 2, which also include the potential roles that they play during primary and secondary infection. Several proteins during primary infection that were identified are related to immunity according to their Gene Ontology (GO) functional annotations. For example, Gch2, which involves in adaptive immunity and responds to interferon (IFN)-γ stimulation, was identified to be a hub protein. Another hub protein in primary infection is Hsp90a.1, which is involved in Fc-γ receptor signaling. It is interesting to see that Fc-γ receptor signaling may play an important role in antigen presentation in primary *C. albicans* infection in zebrafish.

**Table 1:** **Hub proteins identified in the zebrafish intracellular PPI network for primary infection**

**Rank**	**Zebrafish protein**	**Degree**	**GO functional annotation**
**1**	Tfap2a	10	Regulation of cell differentiation
**2**	Gch2	10	Response to interferon γ
**3**	Hsp90a.1	10	Fc-γ receptor signaling
**4**	Acvr1b	9	TGF-β receptor activity
**5**	Btg2	9	Negative regulation of cell proliferation
**6**	Cct5	9	Response to virus
**7**	Clock	9	Circadian rhythm
**8**	Fkbp5	9	Heat shock protein binding
**9**	Fos	9	Innate immune response
**10**	Ncstn	9	T cell proliferation

**Table 2:** **Hub proteins identified in the zebrafish intracellular PPI network for secondary infection**

**Rank**	**Zebrafish protein**	**Degree**	**GO functional annotation**
**1**	Psmd1	16	Antigen processing and presentation of exogenous peptide antigen via MHC class I
**2**	Fos	12	Innate immune response
**3**	Psmd13	11	Antigen processing and presentation of exogenous peptide antigen via MHC class I
**4**	Ndrg1	10	Mast cell activation
**5**	Casp2	10	Regulation of apoptosis
**6**	Gch2	10	Response to interferon-γ
**7**	Ncstn	9	T cell proliferation
**8**	Acvr1b	9	TGF-β receptor activity
**9**	Uba5	9	Protein ubiquitination
**10**	Usp14	8	Regulation of proteasomal protein catabolic process

We further calculated betweenness centrality for each node in both PPI networks. Betweenness measures the number of passing shortest paths through a node [15]. We listed top 10 proteins ranked by betweenness centrality for both primary and secondary infection PPI networks in Table 3. Generally speaking, many hub proteins selected based on node degree during primary infection were also selected based on betweenness centrality. During secondary infection, however, only one hub protein that has a node degree larger than 9 was selected based on betweenness centrality. This is because the PPI network for primary infection is more condensed than the PPI network for the secondary infection. During primary infection, hub protein such as Elavl1, Btg2, Fkbp5, Hug, Hsp90a.1 are also top in the betweenness ranking. Zgc:63606 connects to two hub proteins Elavl1 and Hug, leading to a large betweenness centrality. Similarly, Zgc:153257 is the bridge between two subnetworks, leading to a larger betweenness. Although high in betweenness centrality ranking, these two zebrafish proteins are still unknown in function and may be good candidates for future experiment to verify their relations with immunity. In the case of secondary infection, since the PPI network is more dispersed and contains lots of small networks without a connection to the main network, the betweenness centrality ranking is dominated by the nodes in the main network. Lots of hub proteins with a high degree are not in the top list ranked by betweenness since they are located at the smaller subnetworks that are not connected to the main network, leading to a smaller number of total passing shortest paths through these proteins. On the other hand, despite Tp53, Dhfr and Hspd1 have only a degree of 2, they act as bridges between subnetworks within the main network, leading to a larger betweenness and may potentially be important candidates for further investigation.

**Table 3:** **Proteins identified in the zebrafish intracellular PPI network for primary infection and secondary infection are ranked by betweenness centrality**

	**Primary infection**			**Secondary infection**		
**Rank**	**Zebrafish protein**	**Betweenness centrality**	**Degree**	**Zebrafish protein**	**Betweenness centrality**	**Degree**
1	Elavl1	6048.44	9	Psmd1	16588.53	16
2	Zgc:63606	5972.60	5	Creb1	15020.0	5
3	Btg2	5092.00	9	Psme3	14903.00	4
4	Zgc:153257	5016.00	2	Tp53	14670.00	2
5	Fkbp5	4542.00	9	Cebpb	14334.60	6
6	Hug	4353.72	10	Mdm2	13140.00	3
7	Cul1a	3992.00	3	Casp3a	13080.00	6
8	Hsp90a.1	3642.00	10	Dhfr	13032.00	2
9	LOC563808	3641.05	5	Hspd1	12922.00	2
10	Esr1	2856.00	2	Bcl2	12612.34	4

### Inspection of proteins common to PPI networks for primary and secondary infection

This section focuses on the 20 identified proteins that were common to both primary and secondary infection in our constructed PPI networks. To identify the proteins that play crucial and distinct roles in innate and adaptive immunity, we compared the PPI linkages between primary and secondary infection for each of these common proteins. On the basis of the number of changes in the PPI linkages, these proteins were ranked to identify the top ten proteins whose PPI linkages differed most significantly between primary and secondary infection (Table 4).

**Table 4:** **Zebrafish proteins identified to have the most significant changes in the number of PPI linkages between primary and secondary** ***C. albicans*** **infection**

**Rank**	**Zebrafish protein**	**Primary infection**	**Secondaryinfection**	**Changes**	**Involved biological process**	**Reference**
1	Psmd13	5	12	17	Adaptive immunity	[16]
2	Psmd1	6	11	17	Adaptive immunity	[16]
3	Casp2	5	11	16	Apoptosis	[17]
4	Ncstn	5	11	16	Immune recognition	[18]
5	Wdr82	6	10	16	Unknown	
6	Fos	3	9	12	Innate immunity	
7	Acvr1b	1	8	9	Induction of apoptosis	[19]
					Activation of mast cells	[20]
					Regulation of immunoglobulin	[21]
8	Ampste24	4	4	8	Unknown	
9	Loc553343	3	4	7	Unknown	
10	Hsp90a.1	2	4	6	Apoptosis	[22,23]
					Antigen presentation	[24]

## Discussion

### The TGF-β pathway is involved in the control of the primary and secondary immune response

Acvr1b (ALK4, activin receptor type 1B) was identified to be a hub protein in both primary and secondary infection (Tables 1 and 2). Activins are members of the TGF superfamily that act as local regulators of biological processes and are associated with cell growth and differentiation [19]. Correspondingly, activin is crucial to the control of innate and adaptive immune responses [10].

**Figure 3:**
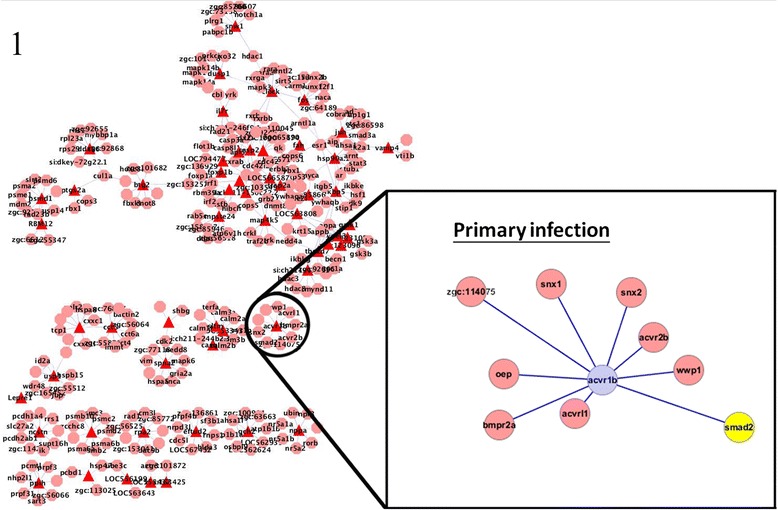
**Interactions of Acvr1b with other proteins in primary infection.** Acvr1b was identified to be a hub protein during both primary and secondary infection. The R-SMAD Smad2 was found to be a key protein in primary infection. The identification of R-SMAD in both primary and secondary infection suggests that the activation of TGF-β signaling requires the SMAD pathway.

**Figure 4:**
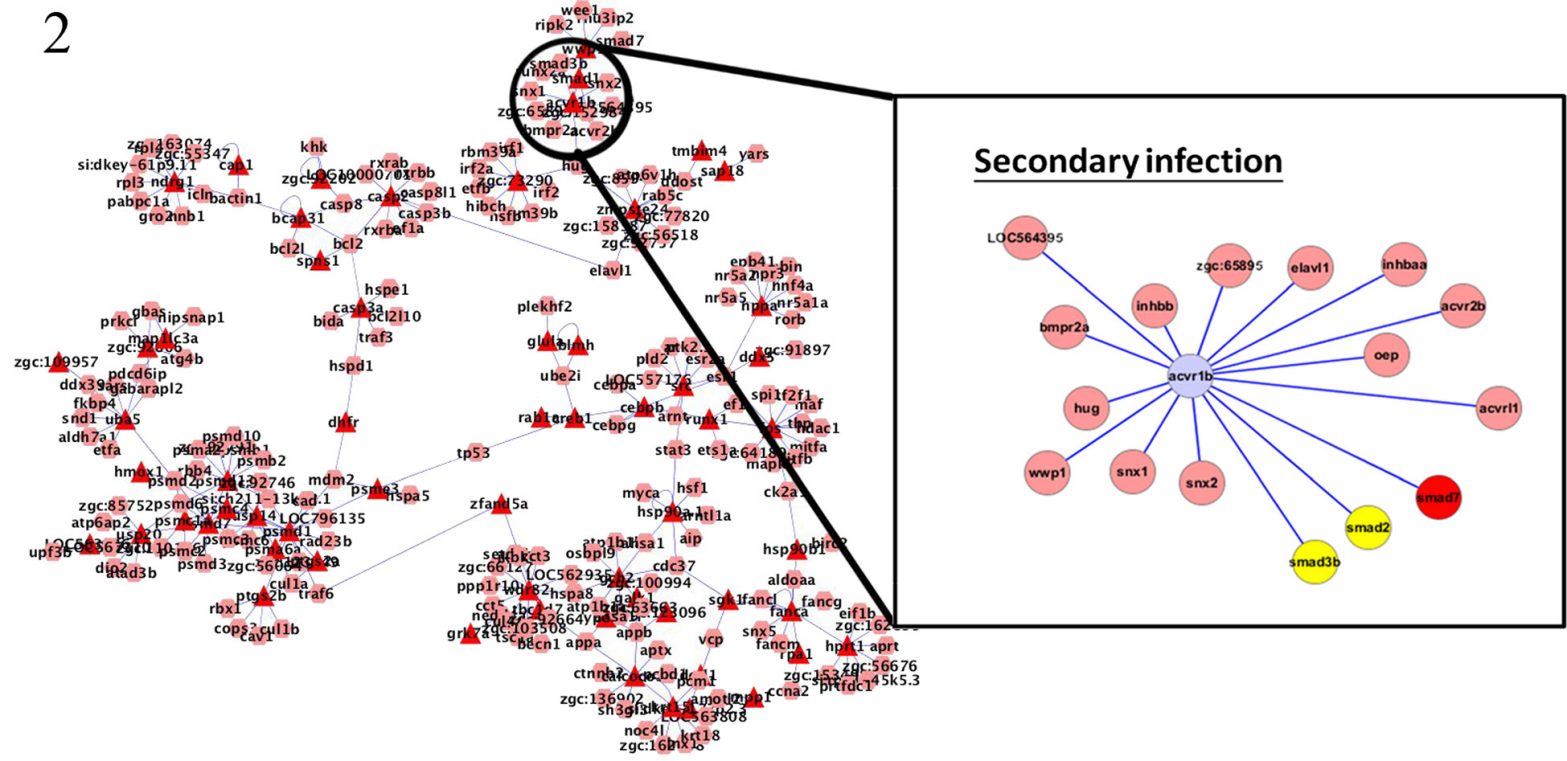
**Interactions of Acvr1b with other proteins in secondary infection.** Acvr1b, Smad2 and Smad3 were found to be important in secondary infection. Smad7, an inhibitory SMAD protein, interacted with Acvr1b in secondary but not primary infection. This disparity suggests that TGF-β signaling may play a role in the control of innate and adaptive immune responses.

To regulate the immune response, TGF-β mediates its effects via SMAD proteins [9]. To clarify the role of TGF-β in the innate and adaptive immune response, we focused on the protein interactions of SMAD proteins and Acvr1b in primary and secondary infection, and compared the differences between initial and recurring infections. In primary infection, the SMAD protein Smad2 was found to interact with Acvr1b; whereas in secondary infection, Smad2, Smad3, and Smad7 were found to interacted with Acvr1b. The SMAD proteins can be divided into three major groups according to their function: receptor-regulated SMADs (R-SMAD), the common mediator SMAD (Co-SMAD), and inhibitory SMADs (I-SMAD) [9]. The R-SMADs Smad2 and Smad3, which, upon phosphorylation, interact with Co-SMAD and translocate to the nucleus [11], were identified as key proteins in primary and secondary infection. However, Smad7, an I-SMAD, was found to interact with Acvr1b during secondary, but not primary, infection (Figures 3 and 4).

**Figure 5:**
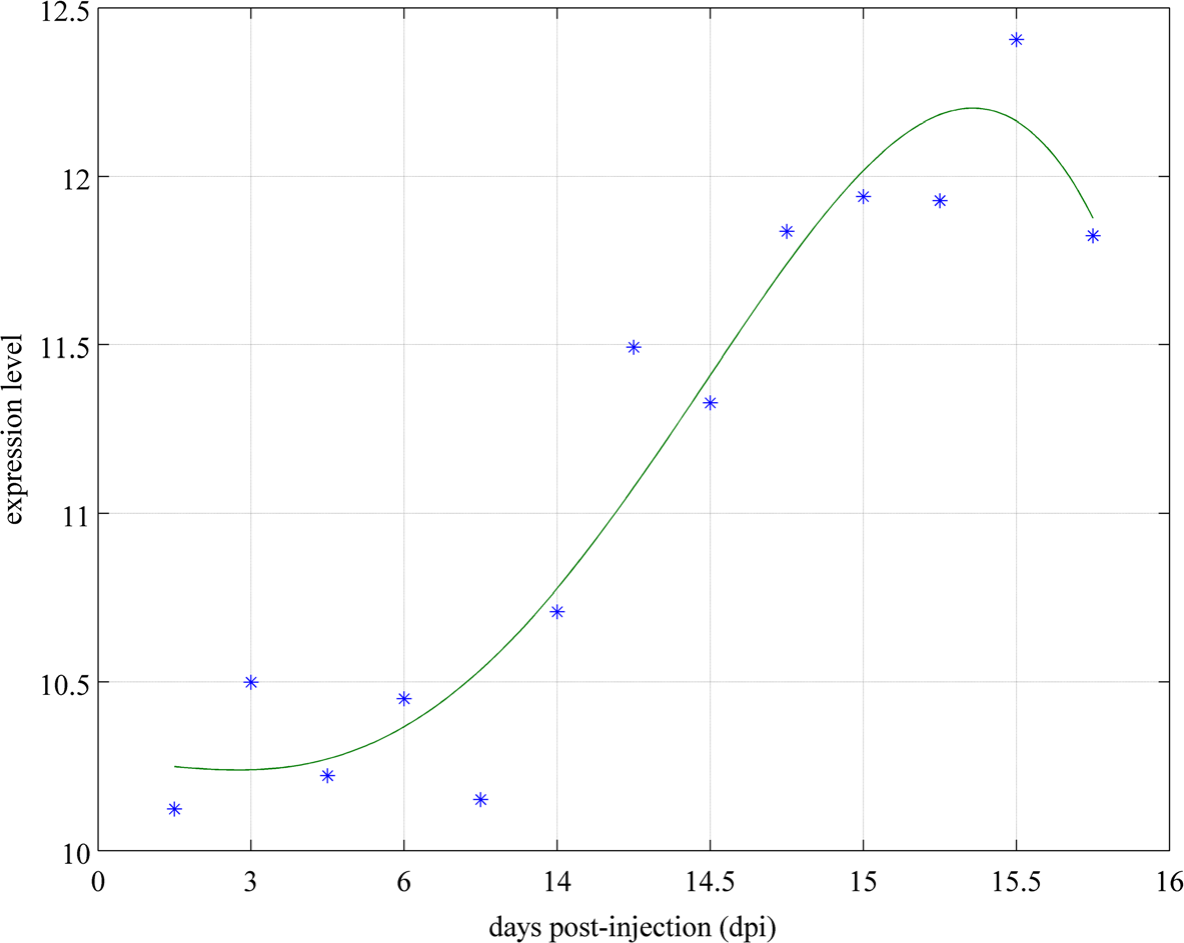
**The expression profile of Smad7 in primary and secondary infection.** Upon comparing the time course of Smad7 expression between the primary and secondary infection, it is clear that Smad7 expression remained stable in primary infection. During secondary infection, however, the level of Smad7 expression increased significantly by an average of 14%. This difference suggests that Smad7 may play a key role in the control of innate and adaptive immune responses.

Smad7 has been reported to play an essential role in the negative regulation of TGF-β signaling by interfering with the binding of TGF-β to type I receptors [25]. Furthermore, we compared the expression profile of Smad7 over time during primary and secondary infection to see if there was a significant change in between infections (Figure 5).

The time course of Smad7 expression showed a significant increasing trend of inhibitory Smad7 expression during secondary infection, suggesting that TGF-β signaling is suppressed in secondary infection relative to primary infection. This is in agreement with previous findings suggesting that Smad7 is involved in the reciprocal inhibition of TGF-β and IFN-γ [26].

In the reciprocal inhibition of TGF-β and IFN-γ, Smad7 is the key component responsible for polarizing responses toward either immunity or tolerance to infection. More specifically, although Smad7 can suppress the TGF-β signaling pathway to initiate infection tolerance, it can also promote immunity triggered through the IFN-γ signaling pathway. This is of interest, as the dual role of Smad7 in determining whether immunity or pathogen tolerance occurs is suggestive of a key mechanism that controls the immune response.

**Figure 6:**
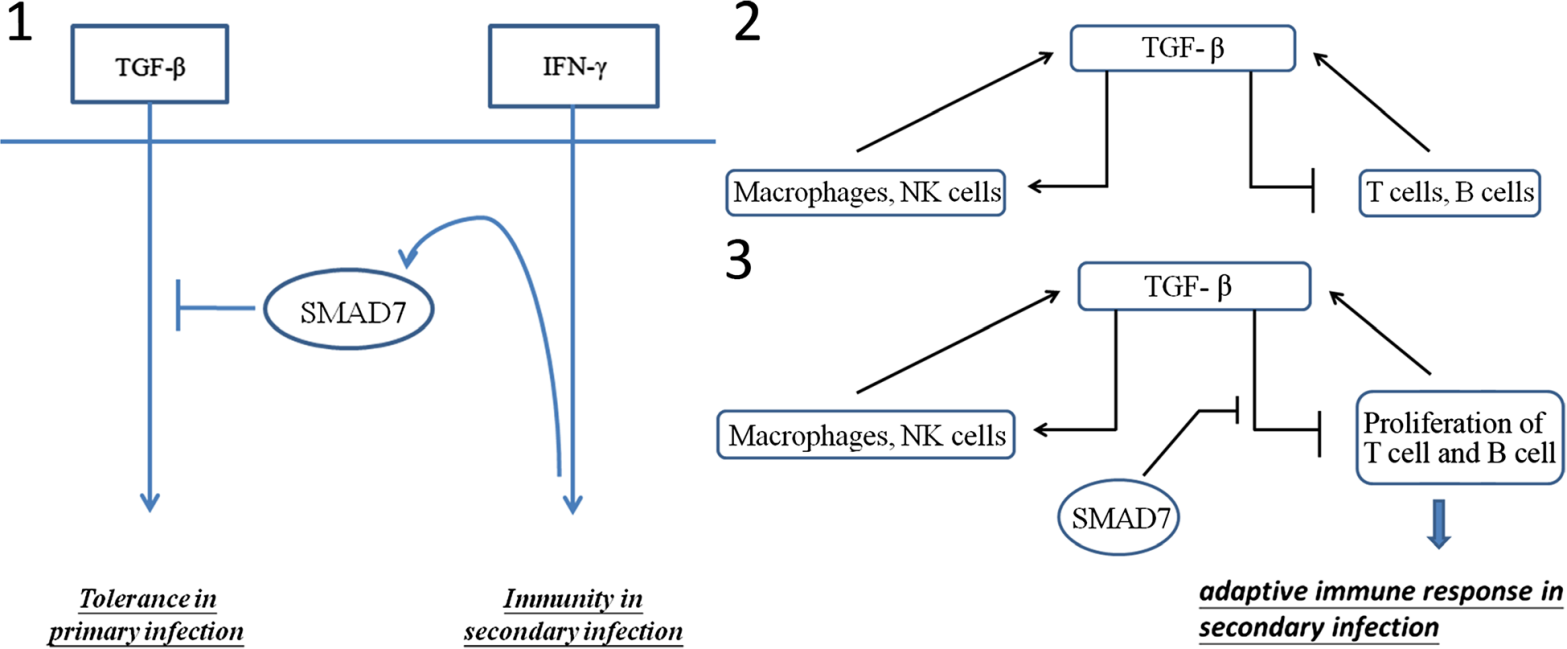
**Smad7 is the key for the regulation of tolerance and immunity.** Figure 6-1: A previous study has reported that Smad7 is a mediator that controls the tolerance and immunity pathways [26]. The time course of Smad7 expression demonstrated a significant increase during secondary infection, suggesting that in zebrafish injected with a higher pathogen dose, the immune response was triggered to defend against the invading pathogen rather than to promote infection tolerance. Figure 6-2: According to a previous study [10], the regulation of TGF-β, the innate immune response, and the adaptive immune response may be considered as a feedback system. Figure 6-3: Smad7 was found to be a key protein during secondary infection. Smad7 attenuates TGF-β-mediated inhibition of cells in the adaptive immune system, resulting in the proliferation of T and B cells that promote host defense by the adaptive immune system challenged with a higher dose of the pathogen.

The gene expression time course in primary and secondary infection showed that Smad7 expression, which is at basal level in primary infection, increased rapidly during secondary infection (Figure 5). The difference in Smad7 expression between the primary and secondary infections may indicate that after initial low-dose infection, the zebrafish immune system was able to tolerate the invading pathogen, thereby shifting the immune response toward infection tolerance. However, in secondary infection with a lethal pathogen dose, increased Smad7 expression suggests that the immune response is triggered to defend against the invading pathogen; thus, the pathway responsible for infection tolerance is inhibited in this phase (Figure 6).

In addition, TGF-β has also been suggested to inhibit the function of inflammatory cells and immune responses [8,27]. The regulation of TGF-β and its relationships with various immune cells are depicted in Figure 6. Under normal conditions, there is a feedback system that characterizes the relationship between the TGF-β pathway, the innate immune response, and the adaptive immune response (Figure 6). Even though immune cells can secrete cytokines that promote TGF-β signaling, TGF-β signaling can inhibit activation of these immune cells, thus acting as a feedback system. Smad7 during secondary infection was found to suppress TGF-β signaling, leading to attenuated inhibition of immune cells (Figure 6). Consequently, the increased proliferation of immune cells such as T and B cells in the adaptive immune response promotes defense against the invading pathogen.

In summary, the identification of Acvr1b in primary and secondary infection suggests that TGF-β signaling is indeed involved in the control of innate and adaptive immune responses. Furthermore, the discovery that Smad7 interacted with Acvr1b only during secondary infection suggests that TGF-β controls immune responses via a SMAD-dependent pathway. Therefore, the control mechanism can be described as a feedback system involving TGF-β signaling and the adaptive immune response (Figure 6).

### The proteasome plays a role in controlling the adaptive immune response

Psmd1 and Psmd13, 26S proteasome regulatory subunits, were identified to be significant primarily during secondary infection. Proteasomal activity has been shown to be related to inflammatory and autoimmune diseases such as systemic lupus erythematosus and rheumatoid arthritis because of its role in activating an anti-apoptotic and pro-inflammatory regulator of cytokine expression [28]. Therefore, the identification of Psmd1 and Psmd13 in our constructed intracellular PPI networks for secondary infection indicates that the proteasome system plays a pivotal role in the zebrafish immune response. Furthermore, the numbers of linkages from primary to secondary infection for both Psmd1 and Psmd13 increased significantly, suggesting that the proteasome is more active during secondary infection, and is therefore more important in the adaptive immune response of zebrafish.

### Regulation of apoptosis in primary and secondary infection

Many of the ten most significant hub proteins discussed above are related to the apoptotic process, as shown in Table 1. Further investigation revealed that apoptosis was activated during primary infection but was inhibited during secondary infection. In our constructed zebrafish intracellular PPI networks, we identified three proteins (Casp2, Acvr1b, Hsp90a.1) that were involved in apoptosis out of the ten hub proteins (Table 4).

**Figure 7:**
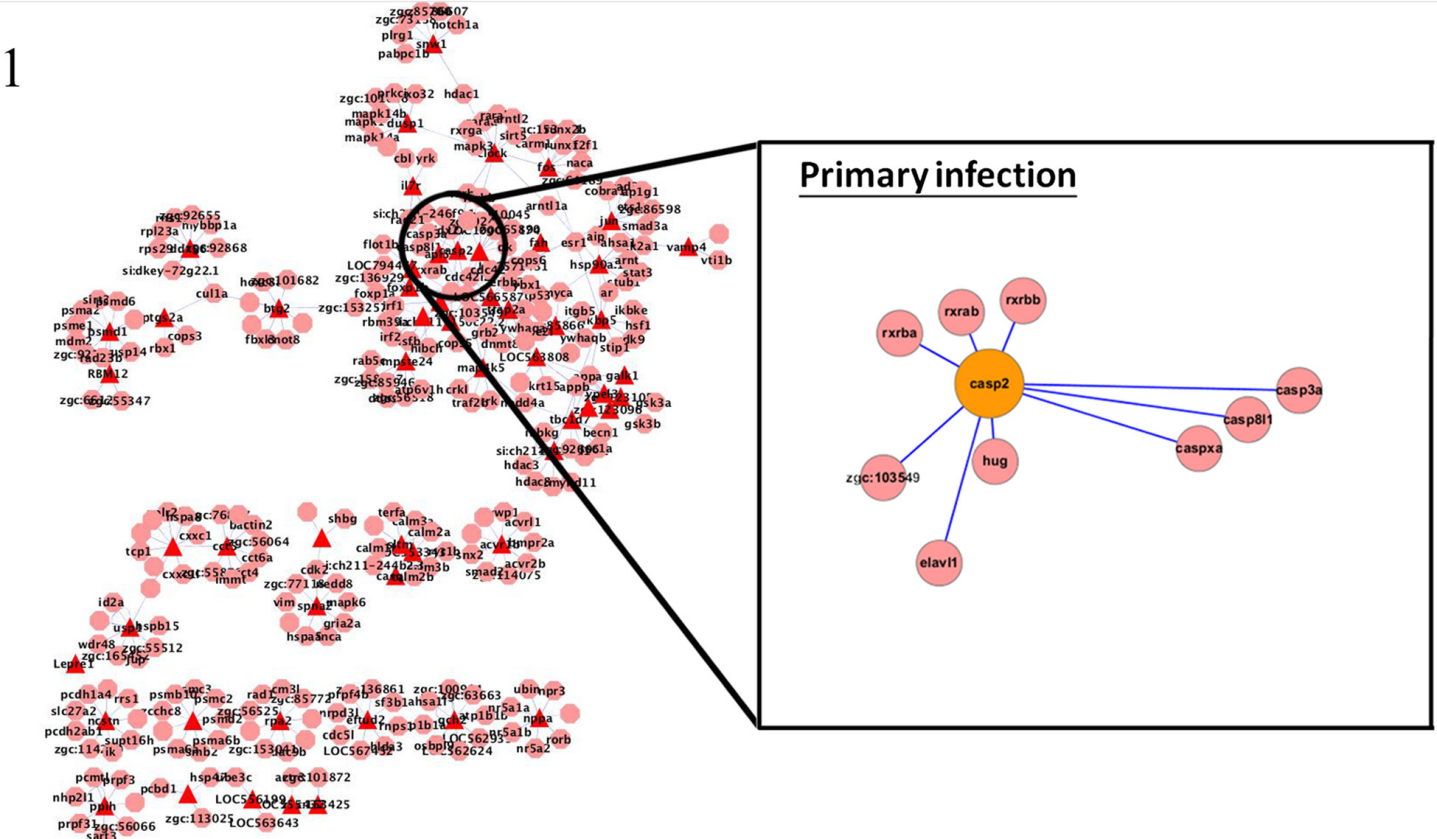
**Protein interactions of caspase 2 in primary infection.** Caspase 2 was identified to be a hub protein during both primary and secondary infection. Bcl2, an anti-apoptotic protein, did only interact with Casp2 during primary infection.

**Figure 8:**
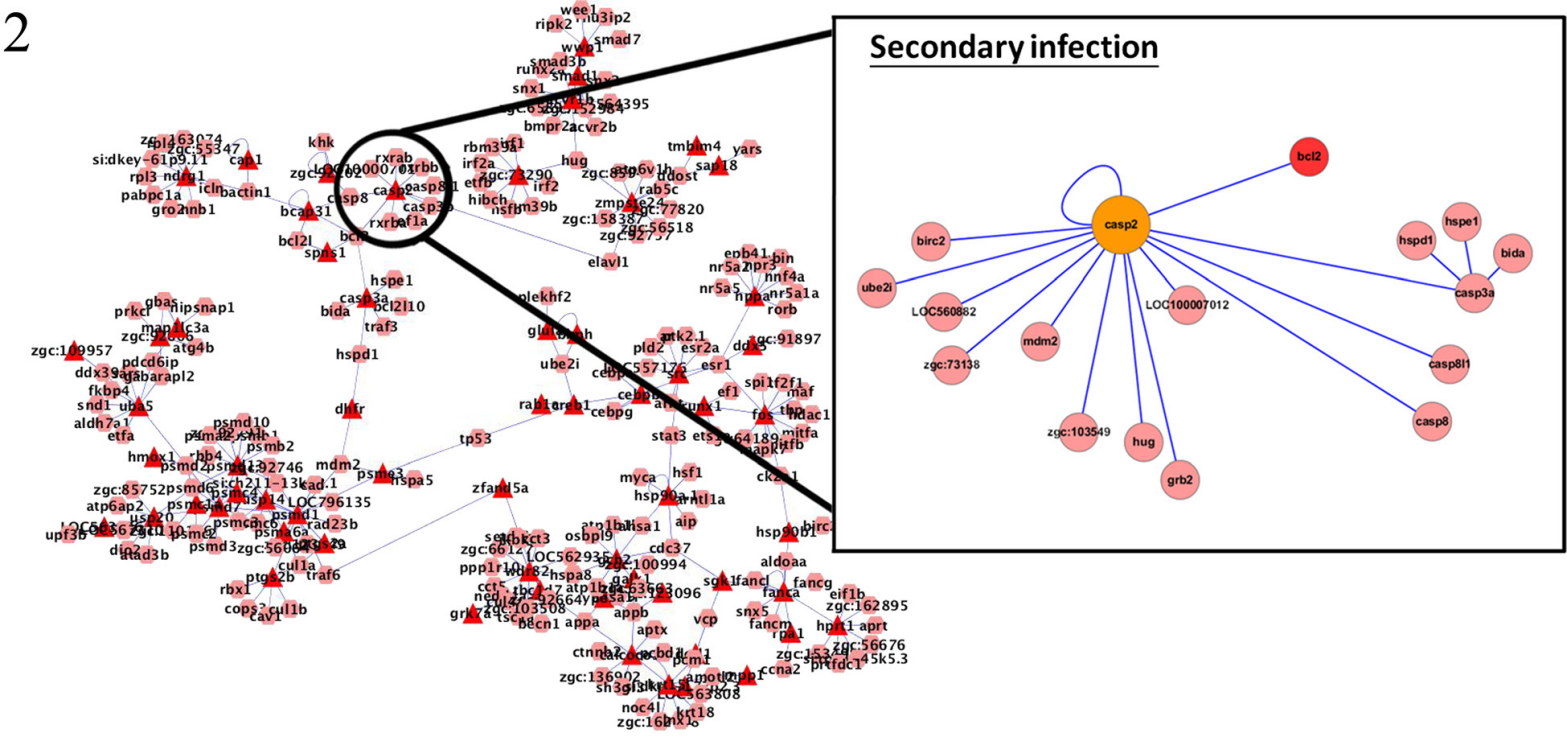
**Protein interactions of caspase 2 in secondary infection.** Caspase 2 was identified to be a hub protein during both primary and secondary infection. Bcl2, an anti-apoptotic protein, only interacted with Casp2 during secondary infection, suggesting that the apoptotic process is inhibited in secondary infection.

For Casp2, the number of linkages increased significantly during secondary infection relative to the number during primary infection. The caspase family of proteins has a dominant role in activating apoptosis [29]. Analysis of Casp2 protein interactions revealed that it interacted with Bcl2 during secondary but not primary infection (Figures 7 and 8). Bcl2 is a member of the Bcl2 family that regulates cell death by inhibiting the apoptotic process [30]. Thus, the finding that Casp2 and Bcl2 interacted during secondary infection suggests that apoptosis is suppressed during secondary infection, in contrast to the induction of apoptosis during primary infection.

Acvr1b, a type 1B activin receptor, has been shown to be related to the apoptotic process in both primary and secondary infection. Activins are members of the TGF-β superfamily and are local regulators of biological processes that are associated with cell growth and differentiation [19]. The TGF-β pathway is also involved in inducing apoptosis and the SMAD family of molecules act as key signal transducers during this apoptotic process [31]. Smad7 protein was found to interact with Acvr1b during secondary but not primary infection (Figures 3 and 4). Smad7 is an inhibitory protein that interferes with the phosphorylation of pathway-restricted SMAD proteins such as Smad2 and SMAD3 by binding to type I receptors [11,32]. Therefore, the interaction between Acvr1b and Smad7 supports our observation that apoptosis is inhibited during secondary *C. albicans* infection.

Hsp90a.1, a heat shock protein, was identified to be a key hub protein in the zebrafish intracellular PPI networks. The number of its linkages increased significantly from primary to secondary infection. Hsp70 and Hsp90 directly interact with proteins regulating the programmed cell death machinery and thus block the apoptotic process [23]. The identification of Hsp90a.1 as an important protein mainly during secondary infection in our constructed network again suggests that apoptosis is inhibited during secondary infection. Furthermore, Hsp90 stabilizes the 26S proteasome (Psmd1 and Psmd13, 26S proteasome proteins that are a part of the ten hub proteins, as listed in Table 4), and thereby enables the cell to remove unwanted or harmful proteins.

In summary, the finding that apoptotic proteins such as Casp2, Acrv1b, and Hsp90a.1 are more prominent during secondary rather than primary infection is intriguing. Increasing evidence supports that apoptosis has a crucial role in innate and adaptive immunity during infection [33-36]. Our results indicate that apoptosis was inhibited in secondary but not primary infection, suggesting that during infection, apoptosis can be adopted as an offensive or defensive strategy by the pathogen or zebrafish, respectively.

### The identification of Ncstn implies a relationship between bacteria- and fungus-induced immune responses

Ncstn, a part of the γ-secretase protein complex, was found to play a significant role during both primary and secondary infection in our constructed PPI networks. Ncstn can generate a peptide epitope that facilitates immune recognition of intracellular mycobacteria with related components of γ-secretase through MHC II-dependent priming of T cells [18]. Such pathogen recognition mechanisms are crucial to adaptive immunity in the host. The identification of Ncstn during *C. albicans* infection of zebrafish suggests that Ncstn responds not only to bacterial infection but also to fungal infection.

**Figure 9:**
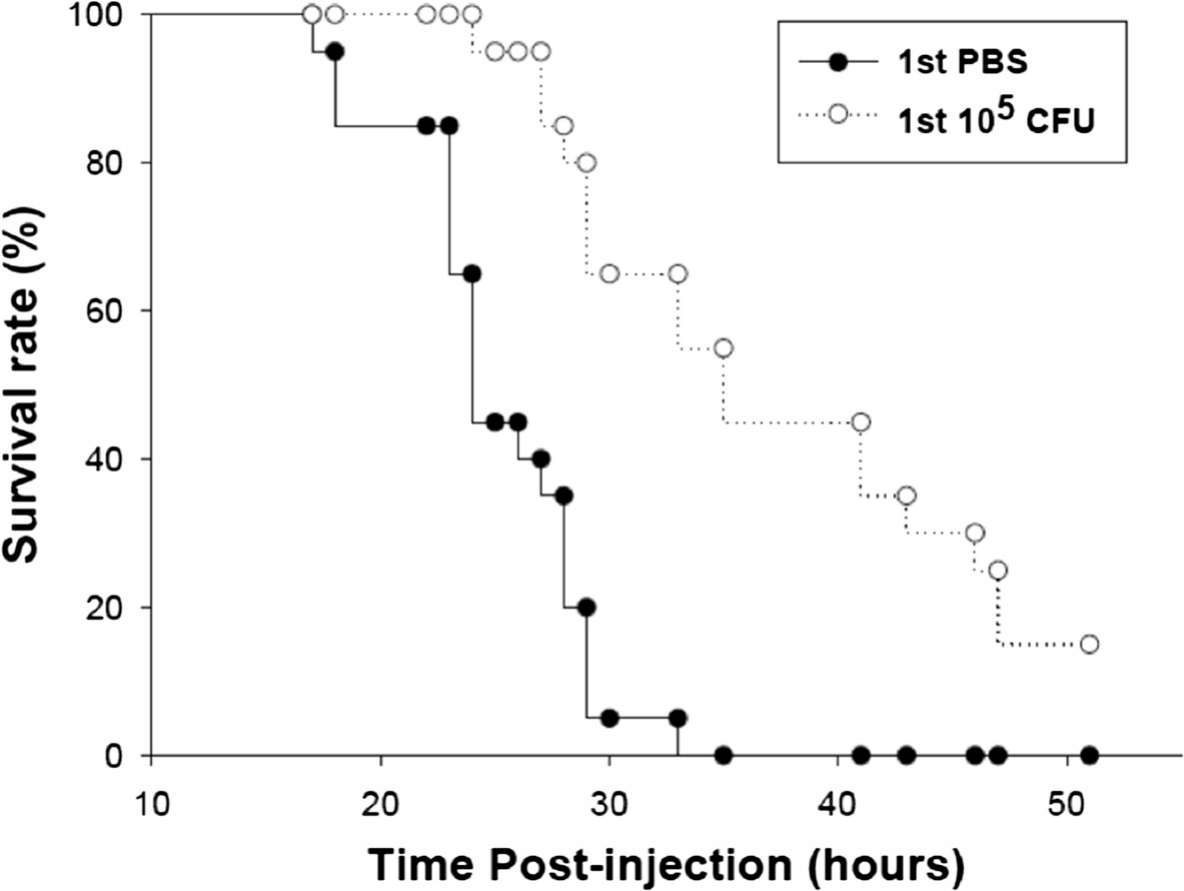
**Zebrafish that have activated the adaptive immune response have a higher survival rate following secondary high-dose infection with** ***C. albicans*** **.** This figure depicts the zebrafish survival rate versus time. In this experiment, zebrafish had been infected with a non-lethal low dose of the live yeast form of *C. albicans* (10^5^ CFU) or injected with PBS. Two weeks later, all fish were infected with a higher dose of *C. albicans* (10^7^ CFU). Zebrafish inoculated with a low dose of *C. albicans* had a survival time longer than that of the PBS group, demonstrating that zebrafish can activate adaptive immunity to defend against repeated *C. albicans* infection.

Taken together, initial investigation of our constructed PPI networks for primary and secondary infection revealed that the immune responses activated after secondary infection are generally stronger. As shown in the *in vivo* experiment, zebrafish that had been infected with 1 × 10^5^ CFU *C. albicans* have a higher survival rate and survive longer after secondary infection with a more lethal *C. albicans* dose (1 × 10^7^ CFU) compared with zebrafish without prior infection (Figure 9). Identification of the aforementioned hub proteins in our constructed zebrafish intracellular PPI networks encouraged us to explore how the zebrafish immune system responds to infection and whether the response differs in primary and secondary infection.

Note that our dynamic modeling approach is not free from errors. False-positive and false-negative interactions in the initial putative PPI network can affect the accuracy of our constructed network. In order to minimize the effect of false-positive interactions, we applied AIC in the last step of network construction to eliminate the false-positive interactions based on model order selection. False-negative interactions are harder to avoid since if a PPI link is missing in the initial putative network, there is no effective method to recover the link. Therefore, we used BioGRID and InParanoid7 database, the most comprehensive PPI database available, to build our initial candidate network. We understand that PPI links may still be missing in BioGRID and InParanoid7. Such error can be improved when more PPI databases are available and can be integrated to form a comprehensive initial putative network.

## Conclusion

Using dynamic modeling and time-course microarray data, we constructed intracellular PPI networks for primary and secondary infection of zebrafish with *C. albicans*. Using these PPI networks, we examined how immune responses in zebrafish are triggered against primary and secondary infection. We identified 341 and 359 intracellular PPIs in the intracellular PPI networks for primary and secondary infection, respectively. Hub proteins of each network were also identified.

By comparing the two constructed PPI networks, the ten proteins with the most significant changes in linkage between primary and secondary infection were determined. These proteins might play crucial roles in the immune response of zebrafish during infection; thus, the biological and molecular processes that these proteins play during primary and secondary infection were investigated.

TGF-β signaling and apoptosis were two of the main functional modules in primary and secondary infection. Smad7, an I-SMAD protein, was found to be important in TGF-β signaling in secondary infection only. Smad7 interferes with R-SMAD phosphorylation and thereby attenuates TGF-β signaling. Therefore, the role of Smad7 in secondary infection suggests that attenuated suppression of immune cells, which enables the adaptive immune response to defend against high-dose secondary infection. We also identified a feedback system that describes the relationship between TGF-β signaling and the immune response.

We discovered several crucial proteins (Casp2, Acvr1b, and Hsp90a.1) associated with apoptosis. As the most significant proteins in secondary infection were involved in the inhibition of apoptosis, the apoptotic process might an important mechanism in the zebrafish immune response against *C. albicans*, particularly during primary infection.

Our initial *in silico* analyses encourage further experimental investigation on the pertinent roles played by apoptosis in the innate and adaptive immune response of zebrafish. We believe that new insights revealed by our work may lead to therapeutic advances and improved design of drugs for the continuous battle against infectious diseases.

## Methods

### Zebrafish strain and maintenance

Male, wild-type AB strain zebrafish were used in the study. Zebrafish were adults approximately 9 months old and weighed 0.33–0.37 g. Fish were maintained in 10 L tanks at 28.5°C under a 14/10 h day/night cycle.

### ***C. albicans*** strain and growth conditions

The SC5314 strain of *C. albicans* was used in this study. A single colony from fresh YPD agar plates (1% yeast extract, 2% peptone, 2% dextrose, 1.5% agar) was inoculated into 5 ml YPD broth and then incubated with shaking at 180 rpm at 30°C for 24 h. Cells were harvested by centrifugation, washed once with sterile PBS or Hank’s balanced salt solution (HBSS), and then resuspended in sterile PBS or HBSS. Suspensions of *C. albicans* cells were diluted with PBS or HBSS and then injected into zebrafish.

### Infection and survival assay

**Figure 10:**
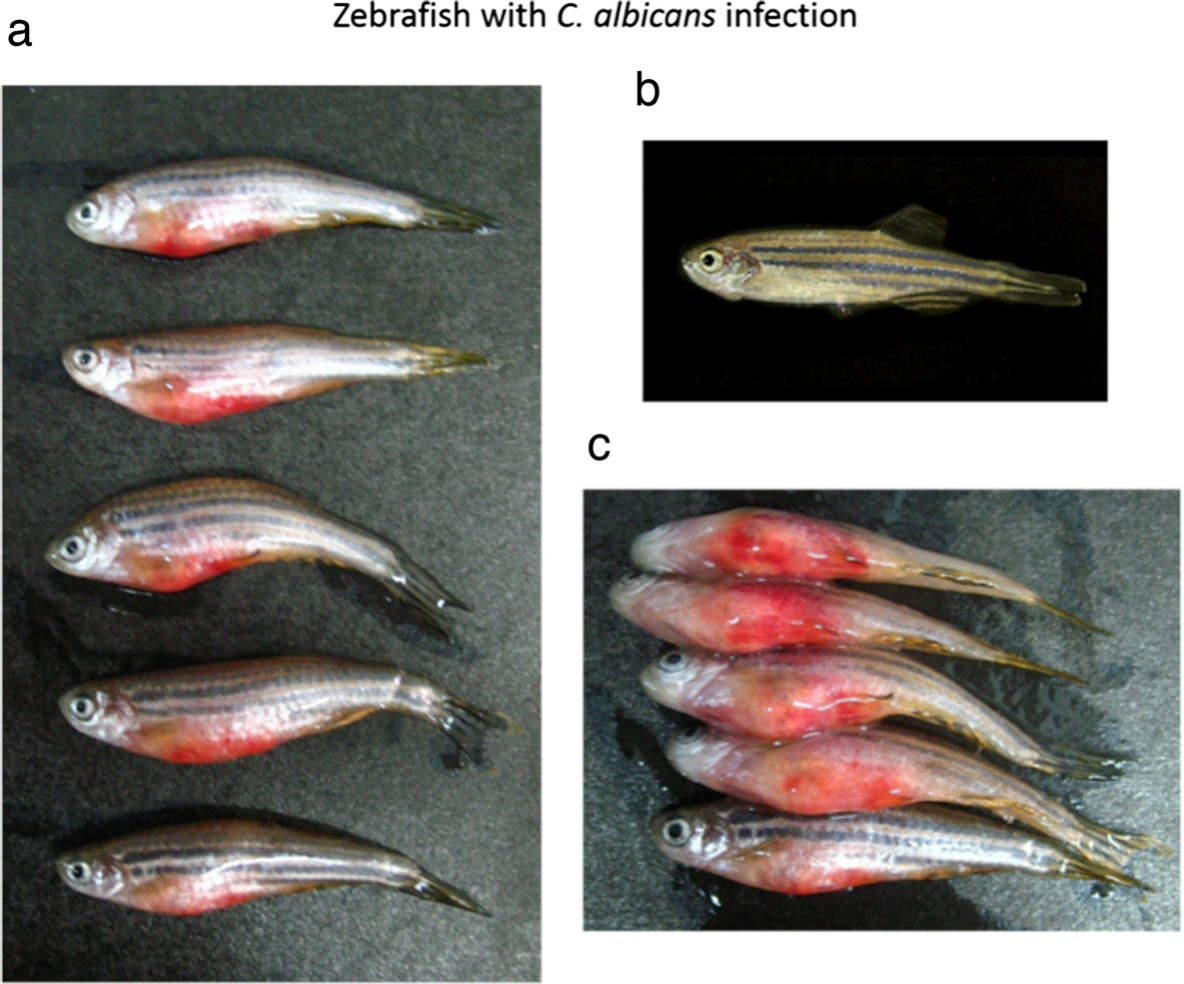
**The phenotypes of infected zebrafish during the secondary infection.** Three typical phenotypes can be observed during the secondary infection, including **a**. bleeding, **b**. ulcer/lesion, and **c**. dropsy/abnormal swelling of ventral position.

Zebrafish were anesthetized by immersion in water containing 0.17 g/ml tricaine (Sigma, USA) and then intraperitoneally injected with 1 × 10^5^ (primary infection) and 1 × 10^7^ (secondary infection) colony-forming units (CFU) of *C. albicans* at day 0 and 14, respectively, by using a 26.5 gauge syringe (Hamilton Syringe 701 N). After infection, fish were immediately transferred to the tanks to recover immediately and kept in separate 10 L tanks maintained with daily water changes. The tanks were housed in an incubator with a 14/10-h day/night cycle at 28.5°C. The fish were closely monitored and mortality was determined every hour. The phenotypes of infected zebrafish, including bleeding, ulcer/lesion, and dropsy/abnormal swelling, were shown in Figure 10.

### Purification of ***C. albicans*** and zebrafish RNA

*C. albicans*-infected zebrafish were immersed in Trizol reagent (Invitrogen, USA) and then ground in liquid nitrogen using a small mortar and pestle. The ground sample was then disrupted by using a MagNALyser System (Roche) with glass beads (cat. no. G8772-100G, Sigma), and then shaken at 5,000 rpm for 15 s. After phase separation by addition of chloroform, the total RNA was purified using an RNeasy Mini Kit (Qiagen, Germany). The purified RNA was quantified at an OD of 260 nm wavelength by using an ND-1000 spectrophotometer (Nanodrop Technology, USA) and the RNA quality was analyzed using a Bioanalyzer 2100 (Agilent Technologies, USA) with a RNA 6000 Nanolabchip kit (Agilent Technologies).

### Microarray experiments

Total RNA (1 μg) was amplified using a Quick-Amp labeling kit (Agilent Technologies), and then labeled with Cy3 (CyDye, PerkinElmer, USA) during the *in vitro* transcription process. For the *C. albicans* and zebrafish arrays, 0.625 and 1.65 μg of Cy3 cRNA, respectively, were fragmented to an average size of approximately 50 to 100 nucleotides by incubation with fragmentation buffer at 60°C for 30 min. The fragmented and labeled cRNA was then hybridized to an oligomicroarray at 60°C for 17 h. The microarrays were washed, then dried by using a nitrogen gun, and then scanned for Cy3 at 535 nm by using an Agilent microarray scanner (Agilent Technologies, USA). Scanned images were analyzed by Feature Extraction 9.5.3 software (Agilent Technologies), and image analysis and normalization software were employed to quantify the signal and background intensities for each feature. Raw data were uploaded onto the NCBI GEO Database (The array data has been uploaded onto NCBI GEO database with accession number: GSE51603).

The time points of the time-course microarray data for the primary infection were 1, 2, 3, 6, and 14 days post-infection (dpi), and those for secondary infection were 14.2, 14.6, 14.12, 14.18, 15, 15.6, 15.12, and 15.18 dpi (Figure 2). Each time point consisted of two replicates with 10 zebrafish in each replica as well as the control group with comparable conditions.

While the microarray dataset presented in this manuscript was newly generated and first reported in this study, it is also a most recent report from a series of pathogen-host interaction studies completed by our research group. Routine validation assays, including histological analysis, have already been performed and reported previously to ensure the quality and reproducibility of our data (ex.[37-39]). In comparison with our published reports that were focusing on the primary infection, the results are highly consistent between the current study and our past findings.

## Additional file

Additional file 1:
**The time profile after ANOVA, PPIN list, PPIN figure and betweenness centrality of Protein-protein interaction network for primary and secondary infection.**
