# Supplementary figures and images for: The role of TGF-β signaling and apoptosis in innate and adaptive immunity in zebrafish: a systems biology approach
Source: BMC Syst Biol. 2014 Oct 24;8:116. doi: 10.1186/s12918-014-0116-0 (PMC4224695; doi:10.1186/s12918-014-0116-0)

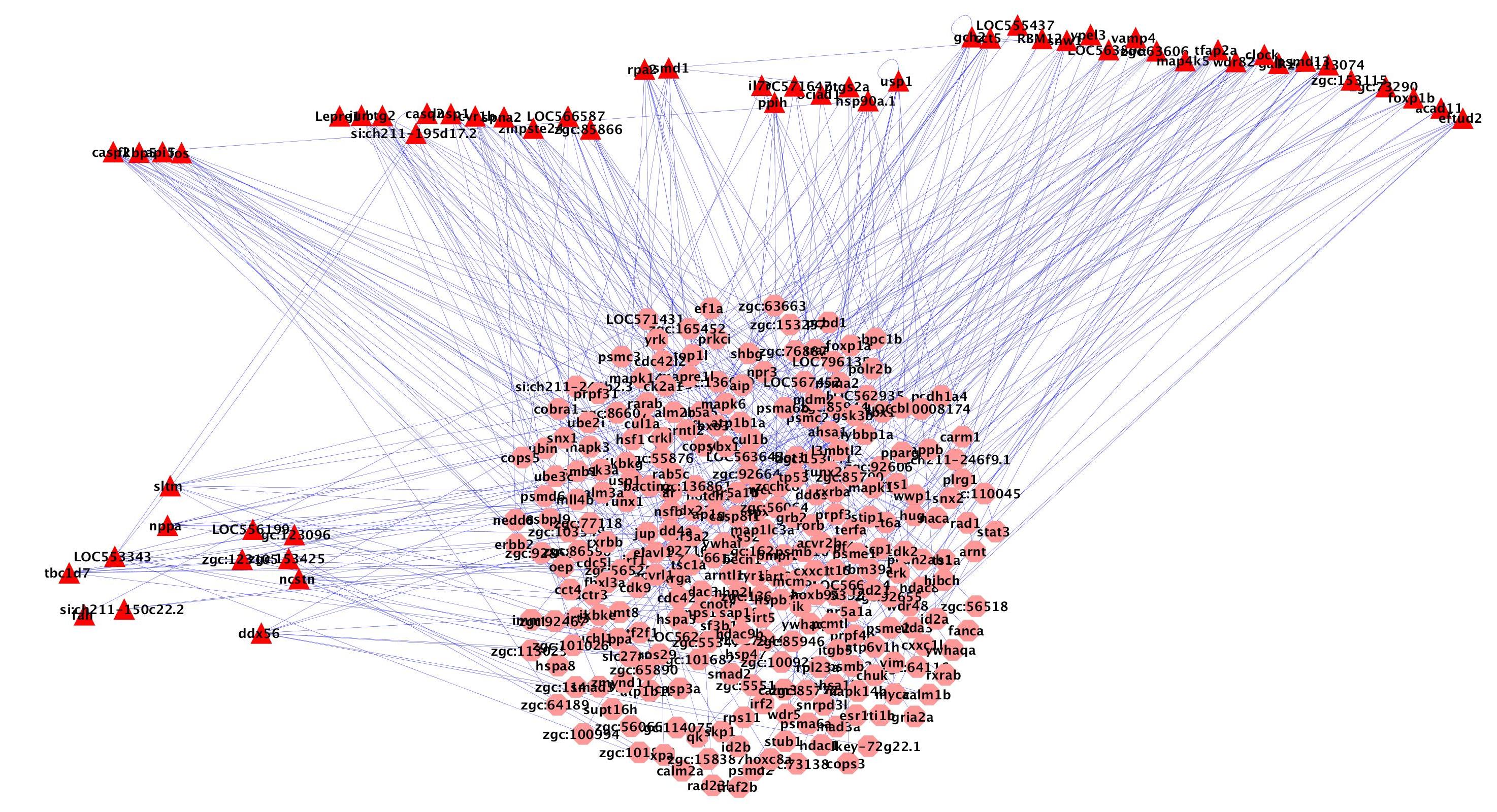

Supplement: Additional file 1: — The time profile after ANOVA, PPIN list, PPIN figure and betweenness centrality of Protein-protein interaction network for primary and secondary infection. [file 12918_2014_116_MOESM1_ESM.zip › Supplementary files/primary infection network.jpg]

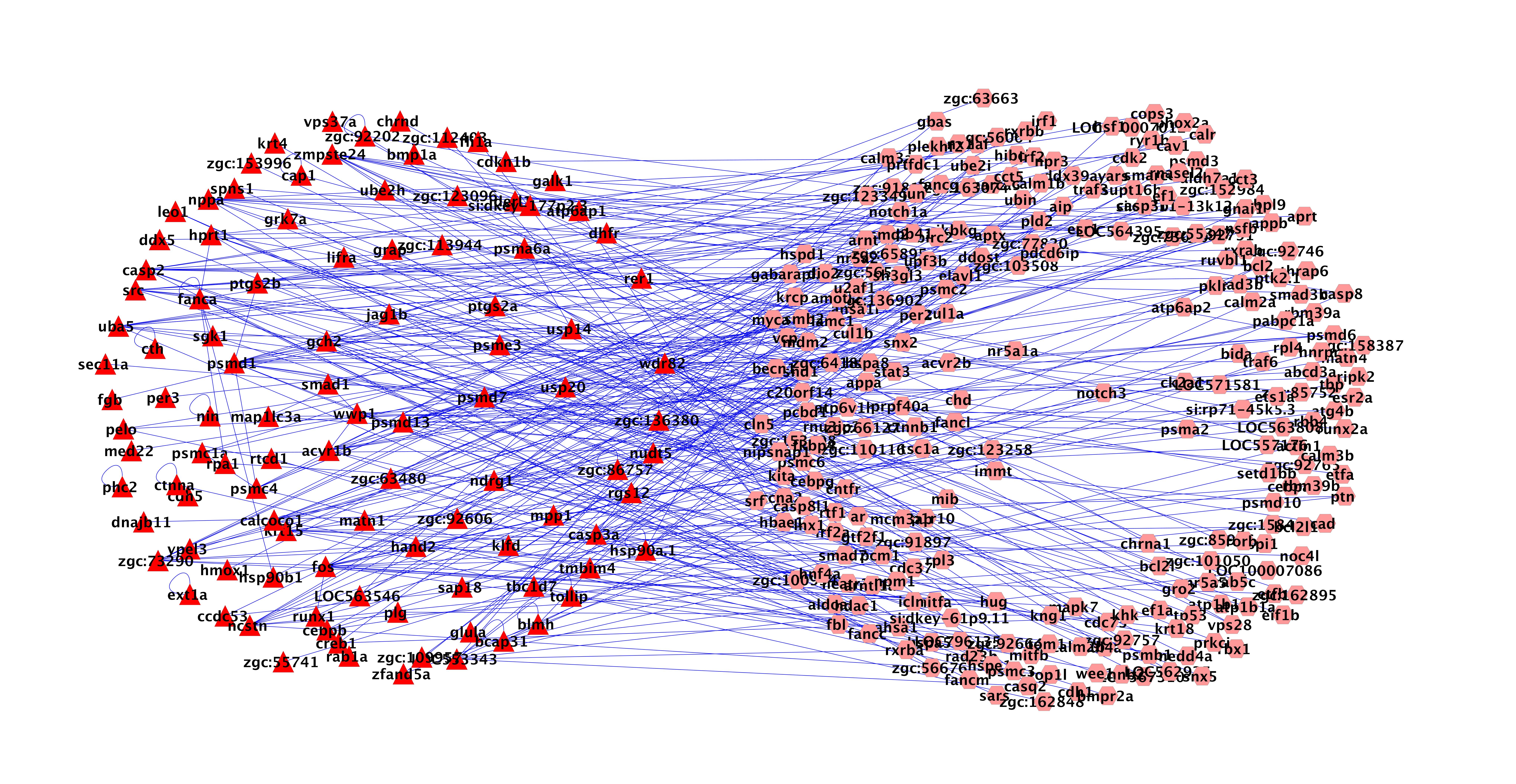

Supplement: Additional file 1: — The time profile after ANOVA, PPIN list, PPIN figure and betweenness centrality of Protein-protein interaction network for primary and secondary infection. [file 12918_2014_116_MOESM1_ESM.zip › Supplementary files/secondary infection network.jpg]
